# Electronic State of Sodium *trans*-[Tetrachloridobis(1*H*-indazole)ruthenate(III)] (NKP-1339) in Tumor, Liver and Kidney Tissue of a SW480-bearing Mouse

**DOI:** 10.1038/srep40966

**Published:** 2017-01-23

**Authors:** Amir Blazevic, Alfred A. Hummer, Petra Heffeter, Walter Berger, Martin Filipits, Giannantonio Cibin, Bernhard K. Keppler, Annette Rompel

**Affiliations:** 1 Universität Wien, Fakultät für Chemie, Institut für Biophysikalische Chemie, Althanstraße 14, 1090 Wien, Austria; 2 Medizinische Universität Wien, Institut für Krebsforschung, “Comprehensive Cancer Center” und Forschungsplattform “Translational Cancer Therapy Research”, Borschkegasse 8a, 1090 Wien, Austria; 3 Diamond Light Source, Didcot, OX11 0DE, United Kingdom; 4 Universität Wien, Fakultät für Chemie, Institut für Anorganische Chemie und Forschungsplattform “Translational Cancer Therapy Research”, Währinger Straße 42, 1090 Wien, Austria

## Abstract

Ruthenium complexes are promising candidates for anticancer agents, especially NKP-1339 (sodium *trans*-[tetrachloridobis(1*H*-indazole)ruthenate(III)]), which is on the edge to clinical applications. The anticancer mechanism seems to be tightly linked to the redox chemistry but despite progress in human clinical trials the *in vivo* Ru oxidation state and the coordination of Ru remains unclear. The Ru-based anticancer drug NKP-1339 was studied applying XANES (Cl K- and Ru L_2,3_-edges) in tumor, kidney and liver tissue of a SW480 bearing mouse. Based on coordination charge and 3D XANES plots containing a series of model compounds as well as pre-edge analysis of the ligand Cl K-edge it is suggested that NKP-1339 remains in its +III oxidation state after 24 hours and at least one of the four chlorido ligands remain covalently bound to the Ru ion showing a biotransformation from Ru^III^N_2_Cl_4_ to Ru^III^Cl_x_(N/O)_6−x_ (X = 1 or 2).

Metallodrugs with cytotoxic properties have a long history as therapeutic agents in oncology. Ruthenium containing complexes have become one of the best developed representatives as new metallodrugs for the treatment of tumors. They show few side effects and resistance^1^,^2^ is unlikely to form against several Ru-based complexes due to the pharmacokinetic and chemical behavior of Ru drugs^3^,^4^. Two of the most recent Ru-containing investigational drug candidates subject to investigation are KP1019 (indazolium *trans*-[tetrachloridobis(1*H*-indazole)ruthenate(III)])^5^ and its sodium analogue NKP-1339 (**7**, sodium *trans*-[tetrachloridobis(1*H*-indazole)ruthenate(III)], Supplementary Figure 1)^6^,^7^. Due to the identical Ru coordination sphere, [tetrachloridobis(1*H*-indazole)ruthenate(III)]^−^, in KP1019 and **7**, it is expected that the main binding partners *in vivo* and their mode of action is highly similar^3^,^8^. Both compounds show remarkable anti-tumor activity in colorectal carcinomas *in vivo* and a variety of primary explanted human tumors *in vitro*^1^,^3^,^8^,^9^. Due to its high water solubility, **7** progressed also to a clinical phase I-IIa study^10^,^11^,^12^. This study is notable for having demonstrated activity of NKP-1339 in cancers unresponsive to prior treatments, such as neuroendocrine tumors of the stomach and small intestine. The current knowledge of the medicinal potential and the mode of action of **7** have been reviewed^7^.

X-ray absorption near-edge spectroscopy (XANES) provides detailed information about the speciation of metal centers such as oxidation state and coordination environment. X-ray absorption spectroscopy (XAS) has been proven to be successful in the study of metal complexes for cancer therapy *in vivo* and *in vitro*^13^,^14^,^15^,^16^,^17^,^18^,^19^,^20^. In contrast to the metal K-edge transitions (1 s → *n*p), the metal L-edges transitions (2p → 4d) are very sensitive to changes in the oxidation state and the electronic environment, such as ligand dissociation and association in biological samples. The soft X-rays needed for the L_2,3_-edges usually have 3–5 times better energy resolution with more intense spectral features^21^,^22^, making L-edges superior to K-edges in terms of oxidation state assignment.

The electronic structures of the tumor-inhibiting compounds KP1019 and NAMI-A (imidazolium *trans*-[tetrachlorido(dimethylsulfoxide)(imidazole)ruthenate(III)])^23^ have been investigated in their solid state applying Cl K-edge and Ru L_3_-edge XANES spectroscopy^24^. Ru K- and L_3_-edge spectra of NAMI-A in the presence of bovine serum albumin (BSA) have been recorded to investigate the oxidation state and the structure of the formed adducts. The coordination environments of the BSA adduct comprises about 60% N and 40% O ligands coordinated to Ru^III^ 20,25. KP1019 was investigated at the Ru K-edge, on the one hand, in citrate saline (CS) buffer (pH 3.5) in the presence of the reducing agent glutathione and, on the other hand, in carbonate buffer (pH 7.4) in a 1:1 mixture with apo-transferrin. XANES analysis revealed replacement of Cl ligands to a different extent in all solutions and suggested a potential interaction with apo-transferrin. KP1019 and **7** were also investigated in tumor and liver tissue with dosages varying between 7.5–40 mg/kg. Ru K-edge spectroscopy did not allow to distinguish between Ru^III^Cl_3_N_2_(O/N) and Ru^II^ClN_2_(O/N)_3_ as assigned coordination modes in the tissue samples^17^. A study on human hepatoma cells treated with KP1019 under cell culture conditions found, besides O/N binding from amine/imine and carboxylato groups of proteins, also indications of S donor atoms binding to Ru^II/III^ that may originate from Cys residues of cytoplasmic enzymes such as cysteine proteases and thioredoxin reductase^19^.

To assign the Ru oxidation state and to understand the electronic distribution of the highly promising tumor inhibiting compound **7** Ru L-edge and Cl K-edge spectroscopy has been performed for the first time on tissue samples. Therefore, one mouse with severe combined immunodeficiency genetic disorder (SCID) bearing the human SW480 adenocarcinoma xenograft was treated with **7** for 24 h. Three different tissue types (tumor = T, liver = L and kidney = K) were investigated (T1, K1, L1) using ligand Cl K-edge and Ru L_2,3_-edge XANES spectroscopy. The XANES investigations of the main detoxification organs in addition to the tumor tissue will address important aspects of the NKP-1339 (Ru^III^Cl_4_N_2_) fate *in vivo*.

## Results and Discussion

### XANES spectra of the model compounds

The XANES region at the Cl K-edge and the Ru L_2,3_-edges were investigated for the following ruthenium (chloride) model compounds (Synthetical protocols cited and structures shown in Supplementary Figure 1), which represent possible Ru coordination and oxidation states *in vivo*: ruthenium(III) acetylacetonate (**1**, with first coordination shell Ru^III^O_6_, Sigma Aldrich, CAS 14284-93-6, 97%)^26^,^27^, hexammineruthenium(III) trichloride (**2**, Ru^III^N_6_, Sigma Aldrich, CAS 14282-91-8, 99%)^28^, (*n*Bu_4_N)_2_[RuCl_3_(ox)(NO)] (**3**, GABU527, Ru^III^Cl_3_NO_2_)^29^, *mer,trans*-aquatrichloridobis(indazole)ruthenium(III) (**4**, KASC003, Ru^III^Cl_3_N_2_O)^30^, *trans,trans*-dichloridotetrakis(indazole)ruthenium(III) chloride (**5**, GABU129, Ru^III^Cl_2_N_4_)^31^, *mer*-trichloridotris(indazole)ruthenium(III) (**6**, GUPL328, Ru^III^Cl_3_N_3_)^32^, sodium *trans*-[tetrachloridobis(1*H*-indazole)ruthenate(III)] (**7**, NKP-1339, Ru^III^Cl_4_N_2_)6, *mer,trans*-trichlorido(dimethylsulfide)bis(indazole)ruthenium(III) (**8**, FLAN005, Ru^III^Cl_3_N_2_S)^33^, tris(bipyridine)ruthenium(II) chloride (**9**, Ru^II^N_6_, Sigma Aldrich, CAS 50525-27-4, 99.95%)^34^, hexammineruthenium(II) dichloride (**10**, Ru^II^N_6_, Sigma Aldrich, CAS 15305-72-3, 99.9%)^28^, *mer*-trichloridotris(ethylphenylsulfide)ruthenium(III) (**11**, FLAN006, Ru^III^Cl_3_S_3_)^35^*, trans,trans*-dichloridotetrakis(indazole)ruthenium(II) (**12**, GABU128, Ru^II^Cl_2_N_4_)^31^ and *trans,trans,trans*-dichloridobis(dimethylsulfide)bis(indazole)ruthenium(II) (**13**, FLAN004, Ru^II^Cl_2_N_2_S_2_)^33^. The Cl K-edge and the Ru L_3_-edge XANES spectra of model compounds **1** to **13** are shown together in Supplementary Figure 2 and the Ru L_2_-edge spectra in Supplementary Figure 3. The edge energies for the Cl K-edge and Ru L_2,3_-edges based on the first maximum in the first derivative of the corresponding normalized XANES spectrum are summarized in Table 1.

The Cl K-edge energy positions of **1** to **13** (Table 1) span a range of 1.4 eV (2824.2 to 2825.6 eV) with **13** (2 Cl covalently bound to Ru^II^) occupying the highest energy (2825.6 eV) and **5** (2 Cl covalently bound to Ru^III^) the lowest energy (2824.8 eV) for the compounds containing bound Cl ligands. **10** (2 Cl^−^ counter anions to a Ru^II^N_6_ coordination sphere) exhibits the lowest edge energy (2824.2 eV). Model compounds with Cl^−^ counter anions (**2**, **9** and **10**) show a higher white line intensity at the Cl K-edge and a steeper rise of the edge (Supplementary Figure 2) after normalization compared to model compounds with covalently bound Cl atoms (**3** to **8** and **11** to **13**). When Cl is covalently bound to an open shell metal, a characteristic pre-edge feature is present in the Cl K-edge spectrum (dashed and dotted spectra in Supplementary Figure 4). This can be attributed to partial mixing of ligand p-orbitals with metal d-orbitals in an otherwise forbidden 1 s → 4d transition^36^,^37^. Therefore, the Cl K-edge spectra in **3** to **8** and **11** to **13** exhibit pre-edge features due to the covalent character of the Ru-Cl bonds, whereas they are missing in **2**, **9** and **10**, where Cl^−^ is the counter anion (solid lines in Supplementary Figure 4). Closer inspection of the Cl K-edge pre-edge features reveals distinct differences between Ru^II^ and Ru^III^ model compounds. Ru^II^ compounds (**12** and **13**) exhibit one pre-edge transition (dotted lines in Supplementary Figure 4), whereas Ru^III^ complexes (**3** to **8**, **11**) show a shoulder or even small separate peak before the pre-edge transition (dashed lines in Supplementary Figure 4). The shoulder has been attributed to an electronic transition to a singly unoccupied molecular orbital^24^. Deconvolutions that resulted from fitting of the Cl K-edge pre-edge region of the octahedral Ru–Cl compounds **3** to **8** and **11** to **13** are given in Supplementary Table 1. The table lists the crystallographic Ru-Cl bond distances as well as the pre-edge energy position, amplitude, FWHM (full width at half maximum), the area for each pre-edge transition and the total area of the pre-edge transitions for each compound. The total pre-edge area increases within the same Ru oxidation state as the number of Cl donor ligands grows, since the total charge donated to the Ru ion is larger. The total area for model compounds **5** (2 Cl covalently bound to Ru^III^), **6** (3 Cl covalently bound to Ru^III^) and **7** (4 Cl covalently bound to Ru^III^) is 1.868 ± 0.019, 1.965 ± 0.014 and 2.176 ± 0.009, respectively. As the Ru oxidation state decreases from Ru^III^ to Ru^II^ the Ru-Cl bond distance becomes longer resulting in decreased orbital overlap between the ligand and metal and thus a lower total area of the pre-edge. The total area decreases from 1.868 ± 0.019 in **5** (2 Cl covalently bound at a average distance of 2.331 Å to Ru^III^) to 1.045 ± 0.057 in **12** (2Cl covalently bound at an average distance of 2.418 Å to Ru^II^).

The Ru L_3_- and L_2_-edge energies of **1** to **13** span a range of 1.5 eV and 1.4 eV, respectively, as compared to 6.1 eV at the Ru K-edge for an almost identical set of model compounds^17^. As reported for the Ru K-edges the edge energies at the Ru L_2,3_-edges increase as the Ru oxidation state changes from Ru^II^ to Ru^III^ and within the same Ru oxidation state the defined edges are shifted to higher energies with increasing electronegativity of the first shell atoms (see Table 1). **1** (Ru^III^O_6_) exhibits the highest edge energy (L_3_: 2842.0 eV L_2_: 2970.1 eV) and **14** (Ru^II^S_2_Cl_2_N_2_) the lowest edge energy (L_3_: 2840.5 eV L_2_: 2968.7 eV). The Ru^III^ model compounds (**1** to **8**, **11**) show a splitting of the Ru L_3_-edge (Supplementary Figure 2) which is missing in Ru^II^ model compounds (**9**, **10**, **12** and **13**), in line with previous observations^24^,^25^. The splitting arises from the transformation of the 4d orbitals into t_2g_ and e_g_ orbitals, in the presence of an octahedral Ru^III^ which has a 4d^5^ electron configuration^38^. In this case the second maximum in the first derivative was used to determine the edge energy position which better correlates with the observed edge energies of the Ru^II^ model compounds.

### XANES spectra of the tissue samples

**Figure 1:**
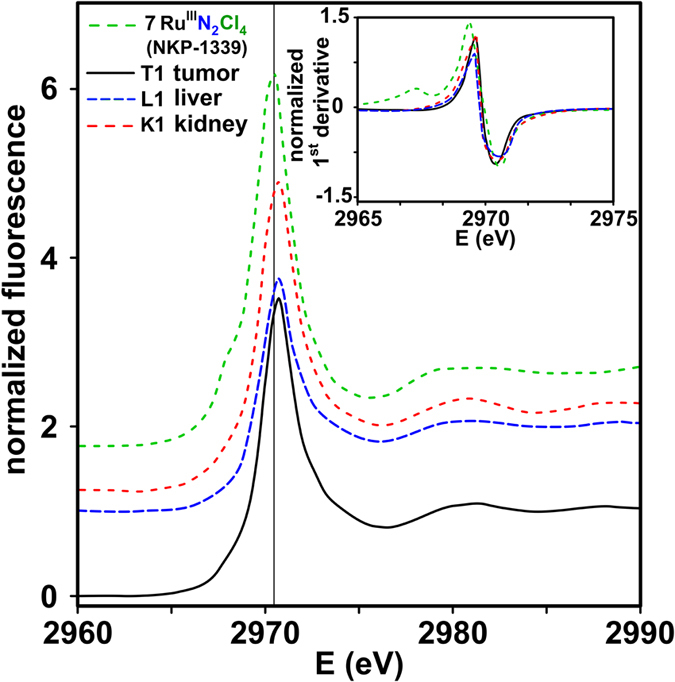
Normalized XANES spectra of the Ru L_2_-edge of tumor, liver and kidney tissue samples. The first derivative is shown in the inset. Spectra are plotted with an arbitrary vertical shift. **7** measured in boron nitride is shown as a reference. A vertical line is inserted that goes through the maximum of **7**.

Tumor, liver and kidney tissue sections were collected from a mouse 24 hours after **7** (40 mg/kg) was administrated intravenously. Data collection of the tissue samples at the Cl K-edge and at the Ru L_2,3_-edges was restricted due to the low Ru concentration and highly abundant Cl^−^ in biological tissue. The high Cl^−^ concentrations showed Cl K-edge signals that were dominated by Cl^−^ contributions, except for the liver sample L1 (*vide infra*), which contained the lowest Cl to Ru ratio and showed a Cl K-edge pre-edge feature. XANES signals were collected at the Ru L_2_-edge for the tissue samples L1, T1 and K1. Figure 1 shows the Ru L_2_-edge and the corresponding first derivative of the tissue samples. The tissue samples show a less pronounced splitting of the edge compared to **7**. Unlike the Ru L_3_-edge, the splitting observed at the Ru L_2_-edge does not necessarily give indications of the Ru oxidation state and can disappear due to e.g. spin-orbit effects^39^. The edge position of all tissue samples increased by 0.3 to 0.4 eV (L1: 2969.8 eV; T1 and K1: 2969.9 eV) compared to **7** (2969.5 eV) suggesting Ru^III^ as the oxidation state and a highly similar coordination sphere around Ru^III^ in the tissue samples.

### Coordination charge and 3D XANES plots

**Figure 2:**
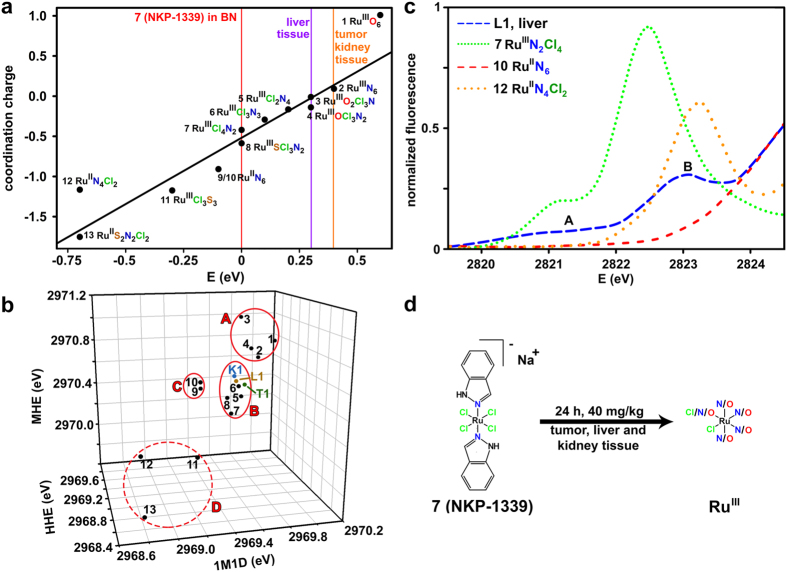
*In vivo* Ru oxidation state. (**a**) Calculated coordination charge η_AR_ according to the Allred–Rochow scale in comparison to the observed edge energies of the XANES spectra in model compounds **1** to **13** (black circles) and the tissue samples (purple and orange lines). The edge energy position was chosen as the first maximum in the first derivative for all model compounds and tissue samples. The edge energy of **7** in boron nitride (BN) is shown as a red line and was set as an arbitrary origin. (**b**) Three dimensional XANES plots based on Ru L_2_-edge maximum height energy (MHE), half height energy (HHE) and energy at the first maximum in the first derivative (1M1D). Model compounds **1** to **13** are shown as black circles and the tissue samples K1, L1 and T1 are shown as colored circles. The plot shows clustering into three distinct regions: Ru^III^ compounds with O ligands (A), Ru^III^ compounds with N/Cl ligands (B) and Ru^II^ compounds with N ligands (C). Region D contain compounds that did not fall into a distinctive cluster. (**c**) The Cl K-edge pre-edge features of **7**, **10**, **12** and L1. Sample L1 show a pre-edge feature with an initial shoulder before an intense pre-edge transition. The L1 spectrum has been smoothed for improved visual appearance. The original spectrum is shown in Supplementary Figure 5. (**d**) Proposed elemental composition and oxidation state for the majority of the Ru centers in the tumor, liver and kidney tissue based on the conforming graphs in (**a**) and (**b**) and the spectrum in (**c**). K1 = kidney 1, L1 = liver 1, T1 = tumor 1.

Discrete spectral features in the XANES region of the well characterized model compounds **1** to **13** can be plotted together and be used as a basis to assign the oxidation state and coordination mode of Ru centers in tumor, kidney and liver tissue. In Table 1 the coordination charge and the corresponding Ru L_2_-edge energy are listed for **1** to **13**, which was plotted against each other in Fig. 2a 17,29. A straight line was regressed with a coefficient of determination *R*^2^ = 0.95, demonstrating a linear correlation between the coordination charge and the edge energy positions. The edge energy of **7** in boron nitride (BN) was set as an arbitrary origin. Ru^III^ compounds containing S and Ru^II^ compounds are found on the lower energy side, whereas Ru^III^ compounds containing N/O are on the higher energy side. The positioning of the tissue samples show a 25% increase in edge energy (0.3–0.4 eV) when comparing to the entire model compound energy range (1.4 eV) at the Ru L_2_-edge (Table 1). At the Ru K-edge the same tissue samples showed an 18% increase in edge energy among a very similar set of model compounds^17^. In terms of Ru L_2_-edge energy position the tissue samples place themselves between **2** (Ru^III^N_6_) and **3** (Ru^III^O_2_Cl_3_N).

Recently the concept of three dimensional XANES plots^40^ was successfully introduced. Key XANES parameters present in model compounds and biological samples, such as edge energies of discrete spectral features and white line intensities are plotted against each other and are used for prediction of oxidation states and coordination spheres in unknown samples. The three dimensional plot in Fig. 2b is based on energies taken from the first maximum in the first derivative, the maximum height and the half height given in Table 1 for the Ru L_2_-edge in order to better separate the Ru oxidation states. 3D-plots with the FWHM and the white line height were also considered in separate graphs but did not generate clusters (the plot including the FWHM is shown in Supplementary Figure 6). The three dimensional plot in Fig. 2b illustrates separation of the model compounds into three different clusters (A-C in Fig. 2b): Ru^III^ compounds with O ligands (A), Ru^III^ compounds with N/Cl ligands (B) and Ru^II^ compounds containing six N ligands (C). Compounds **11** to **13** (Ru^III^S_3_Cl_3_, Ru^II^N_4_Cl_2_ and Ru^II^S_2_Cl_2_N_2_, respectively) were unable to form a distinctive cluster (region D). The tissue samples fall into the upper end of the B cluster (Ru^III^ compounds with N/Cl ligands) extending towards the A cluster (Ru^III^ compounds with O ligands).

The coordination charge correlation and the 3D XANES plot both suggest the presence of Ru^III^ in the tissue samples, which is in good agreement with the observed Cl K-edge pre-edge features of sample L1. Based on earlier findings^24^ and the herein investigated model compounds, the shoulder (A in Fig. 2c) before the pre-edge feature (B in Fig. 2c) indicates presence of Ru^III^. Conclusions of the Ru oxidation state based on the interpretation of the Cl K-edge pre edge features must be drawn with care due to the low quality tissue data (Supplementary Figure 5). However, the existence of a Cl K-edge pre-edge peak indicates that at least one Cl ligand remains covalently bound to Ru (under the assumption that no other Cl-metal open-shell compound is present in L1).

Calculations taking into account the shifts in energy that different ligands and oxidation states induce in the coordination charge correlation confirm that only Ru^III^ is possible for the observed tissue edge energies. The replacement of one Cl ligand by one N ligand in the set of model compounds gives an edge energy shift of approximately 0.1 eV (Table 1). Model compounds **6** (Ru^III^Cl_3_N_3_) and **5** (Ru^III^Cl_2_N_4_) increase by 0.1 and 0.2 eV, respectively compared to **7** (Ru^III^Cl_4_N_2_). The replacement of one N ligand with one O ligand gives no detectable change in the edge energy since both **3** (Ru^III^O_2_Cl_3_N) and **4** (Ru^III^OCl_3_N_2_) exhibit Ru L_2_-edge energies at 2969.8 eV. A change in the oxidation from Ru^II^ to Ru^III^ gives an average shift of 0.7 eV when comparing the edge energy differences between compounds **2** (Ru^III^N_6_, 2969.9 eV) and **9** (Ru^II^N_6_, 2969.4 eV) and **5** (Ru^III^N_4_Cl_2_, 2969.7 eV) and **12** (Ru^II^N_4_Cl_2_, 2968.8 eV). Based on these findings, a Ru^II^ oxidation state with a high number of O/N ligands such as Ru^II^O_5_Cl cannot reach the lowest edge energy observed of at least 2969.8 eV for liver tissue and must therefore be ruled out. However this study cannot rule out S coordinated to Ru(III) in the tissue samples.

The most likely cause for the observed 0.3–0.4 eV shift in all tissue samples compared to solid **7** can be attributed to the replacement of Cl with N and/or O atoms from e.g. amino acids and aqua ligands, respectively (Fig. 2d). The chlorido donors bound to Ru in the metabolites do not necessarily need to come from the parent drug as they can be the result of ligand-exchange reactions with the medium. Ligand exchange has recently been supported by X-ray structure studies^41^ of the highly similar KP1019 bound to human serum albumin, one of the most likely transport proteins^42^,^43^. Two octahedral Ru sites were found with first shell N donor atoms arising from the amino acids (Ru1: His146; Ru2: Lys199 and His242)^41^.

## Conclusions

The complex *trans*-[tetrachloridobis(1*H*-indazole)ruthenate-(III)] (NKP-1339) was used to treat a SW480-bearing mouse of which tumor, liver and kidney tissues were investigated by XANES (Cl K-edge and Ru L_2,3_-edges) and to resolve the Ru oxidation state in target tissue and in the main detoxification organs. The combined XANES analysis from all model compounds and tissue samples suggest a biotransformation of NKP-1339 in all tissues from Ru^III^N_4_Cl_2_ to Ru^III^Cl_x_(N/O)_6−x_ (X = 1 or 2). The presence of Ru^II^ species at his time point (24 h after treatment) can be ruled out.

## Methods

### Application scheme mouse experiments

Citrate saline buffer was prepared by adding 0.42 g of anhydrous citric acid (Sigma Aldrich, 77-92-9, ≥99.5%) to a 400 ml sterile normal saline (0.9% w/v NaCl, Sigma Aldrich, CAS 7647-14-5, ≥99.5%) solution. The solution was stirred until the citric acid dissolved completely (solution A). A second solution was prepared by adding 0.59 g of sodium citrate dihydrate (Sigma Aldrich, CAS 6132-04-3, ≥99%) to a 400 ml sterile normal saline (0.9% w/v NaCl) solution and stirring until everything dissolves. 156 ml of the first solution was combined with 44 ml of the second solution. The resulting CS buffer was adjusted to pH 3.5. Sodium *trans*-[tetrachloridobis(1*H*-indazole)ruthenate(III)] (NKP**-**1339) was prepared as previously reported^6^. CB-17 SCID mice bearing the human xenograft adenocarcinoma cell line SW480 (1 × 10^6^ cells injected subcutaneously into the right flank) were treated on day 21 with NKP-1339. NKP-1339 was dissolved in 5 mM sterile-filtered CS buffer and was applied once intravenously at a dosage of 40 mg/kg. The tissue samples were collected 24 h after the drug administration, flash frozen in liquid nitrogen and kept in −80 °C until the XAS experiments took place 2.5 years later. This batch of samples has also been used to collect the data published in ref. 17 making the performed XAS experiments really complementary. The experiments and methods were done according to the regulations and under the approval of the Ethics Committee for the Care and Use of Laboratory Animals at the Medical University Vienna (proposal number BMWF-66.009/0084-II/3b/2013), the U.S. Public Health Service Policy on Human Care and Use of Laboratory Animals as well as the United Kingdom Coordinating Committee on Cancer Prevention Research’s Guidelines for the Welfare of Animals in Experimental Neoplasia.

### XAS sample preparation

Compounds **1** to **13** were diluted in BN (Sigma Aldrich, CAS 10043-11-5, 99.5%) and held in place with conducting (graphite) tape such that the radiation was incident at an angle of 45° or placed in aluminum holders and sealed with Ultralene foil. The Kapton foil was removed on one side prior to the measurement. The BN preparations were prepared for a calculated absorption of about 1 absorbance unit according to standard methods^44^. The tissue samples were placed in aluminum sample holders and sealed with Kapton foil on both sides.

### XAS experimental setup

XAS experiments were performed at beamline B18 at Diamond Light Source, UK. The ring energy was 3.0 GeV and the ring current 200 mA. The beamline was equipped with a Si(111) monochromator. Higher-energy harmonics were rejected using two Ni-coated Si mirrors at 10 mrad incident angle. The absolute energy calibration was performed using ruthenium powder (Sigma Aldrich, CAS 7440-18-8, 99.9%). The edge position was determined over the first maximum in the first derivative and used for the energy calibration. Spectra were collected in total electron yield, fluorescence and transmission mode for the model compounds and fluorescence mode for the tissue samples. A silicon drift detector was used for the measurements in fluorescence mode. XANES was collected from 2700 to 3100 eV in continuous scan mode with a step size of 0.1 eV. On the tissue samples 70 to 90 scans per sample were collected. The spectra of the model compounds are the average of 3 scans. All tissue samples were collected at 77 K in a “6-way cross” vessel cryostat.

### XANES analysis

Initially careful radiation damage studies were performed by studying spectra collected at different time intervals. The pre-edge background was removed by a linear approximation in the range of −30 eV to −250 eV before the Cl K-edge. Spectra were normalized by fitting a linear spline function to the post-edge region and normalizing the spline to 1.0 at 2860 eV^24^. The edge position was determined as the maximum in the first derivative of the spectrum (inflection point of the steeply rising edge). However, at the Ru L_3_-edge the edge position was determined as the second maximum in the first derivative of the spectrum for all Ru^III^ model compounds due to the splitting observed. For the construction of the 3D XANES plot, additional edge energies were taken at the maximum and half height of the Ru L_2_-edge (see Table 1). The coordination charge and the construction of the coordination charge versus edge plot was constructed in the same way as previously reported^17^. The Cl K-edge pre-edge peaks were fit with Fityk using pseudo-Voigt functions. The number of features in the second derivative determined the number of peaks to fit in the pre-edge and edge regions. In the edge region, an arctangent function was fit for the increase in continuum absorption. Fits were done in the energy range 2818−2825 eV. Spectra in Figs 1 and 2c were smoothed for improved visual appearance in ATHENA using the boxcar average algorithm.

## Additional Information

**How to cite this article**: Blazevic, A. *et al*. Electronic State of Sodium *trans*-[Tetrachloridobis(1*H*-indazole)ruthenate(III)] (NKP-1339) in Tumor, Liver and Kidney Tissue of a SW480-bearing Mouse. *Sci. Rep.* **7**, 40966; doi: 10.1038/srep40966 (2017).

**Publisher's note:** Springer Nature remains neutral with regard to jurisdictional claims in published maps and institutional affiliations.

## Supplementary Material

Supplementary Information

## Figures and Tables

**Table 1:** Edge energies and calculated coordination charges of model compounds 1 to 13 (all octahedral), kidney, liver and tumor tissue.

Compound	Ru first shell	Cl-K PEE (eV)^a^	Cl-K 1M1D (eV)^b^	Ru *η*_AR_	Ru-L_3_ 1M1D (eV)^b^	∆ E to 7	Ru-L_2_ 1M1D (eV)^b^	∆ E to 7	Ru-L_2_ HHE (eV)^c^	Ru-L_2_ MHE (eV)^d^	Ru-L_2_ FWHM (eV)^e^	Ru-L_2_ WLH^f^
**1**	Ru^III^O_6_	n/a	n/a	1.008	2842.0	0.5	2970.1	0.6	2969.6	2970.8	2.6	3.96
**2**	Ru^III^N_6_	n/a	2824.4	0.09	2841.9	0.4	2969.9	0.4	2969.5	2970.6	2.7	3.92
**3**	Ru^III^O_2_Cl_3_N	2821.7	2825.1	−0.012	2841.8	0.3	2969.8	0.3	2969.8	2971.4	2.8	3.96
**4**	Ru^III^OCl_3_N_2_	2821.9	2825.2	−0.141	2841.8	0.3	2969.8	0.3	2969.4	2970.9	2.9	3.95
**5**	Ru^III^Cl_2_N_4_	2822.1	2824.8	−0.166	2841.7	0.2	2969.7	0.2	2969.2	2970.4	2.6	4.10
**6**	Ru^III^Cl_3_N_3_	2822.1	2825.4	−0.294	2841.6	0.1	2969.6	0.1	2969.1	2970.6	2.7	4.11
**7**	Ru^III^Cl_4_N_2_	2822.0	2825.5	−0.422	2841.5	0.0	2969.5	0.0	2969.1	2970.2	2.5	4.12
**8**	Ru^III^SCl_3_N_2_	2821.7	2824.9	−0.588	2841.3	−0.2	2969.5	0.0	2969.2	2970.2	2.6	3.69
**9**	Ru^II^N_6_	n/a	2824.3	−0.91	2841.1	−0.4	2969.4	−0.1	2969.2	2970.5	2.8	3.90
**10**	Ru^II^N_6_	n/a	2824.2	−0.91	2841.1	−0.4	2969.4	−0.1	2969.0	2970.6	3.6	3.93
**11**	Ru^III^S_3_Cl_3_	2821.7	2824.9	−1.176	2841.0	−0.5	2969.2	−0.3	2968.7	2970.1	2.5	3.74
**12**	Ru^II^N_4_Cl_2_	2822.6	2825.1	−1.166	2840.7	−0.8	2968.8	−0.7	2968.7	2970.4	3.1	3.68
**13**	Ru^II^S_2_Cl_2_N_2_	2822.7	2825.6	−1.754	2840.5	−1.0	2968.7	−0.8	2968.6	2969.7	2.6	3.69
K1^g^							2969.9	0.4	2969.3	2970.6	2.4	3.77
L1^g^		2822.5	2825.0				2969.8	0.3	2969.2	2970.5	2.5	3.26
T1^g^							2969.9	0.4	2969.2	2970.4	2.4	3.79

^a^PEE = pre-edge energy determined at the first maximum in the first derivative in the pre-edge region.

^b^1M1D = edge energy position determined at first maximum in the first derivative. At the Ru L_3_-edge the second maximum in the first derivative was used to determine the edge energy position for all Ru^III^ compounds.

^c^HHE = edge energy position determined at half height energy.

^d^MHE = edge energy position determined at maximum height energy.

^e^FWHM = full width at half maximum.

^f^WLH = white line height.

^g^Tissue samples, K1 = kidney 1, K2 = kidney 2, L1 = liver 1, T1 = tumor 1.

Coordination charges (η_ar_) are calculated according to the Allred–Rochow scale.
