# Supplementary material for: Electronic State of Sodium trans-[Tetrachloridobis(1H-indazole)ruthenate(III)] (NKP-1339) in Tumor, Liver and Kidney Tissue of a SW480-bearing Mouse
Source: Sci Rep. 2017 Jan 23;7:40966. doi: 10.1038/srep40966 (PMC5256101; doi:10.1038/srep40966)
Supplement: Supplementary Information [file srep40966-s1.pdf]

Supplementary Information

Electronic State of Sodium *trans* [Tetrachloridobis(1*H*-indazole)ruthenate(III)] (NKP-1339) in Tumor, Liver and Kidney Tissue of a SW480-bearing Mouse

Amir Blazevic[1], Alfred A. Hummer[1], Petra Heffeter[2], Walter Berger[2], Martin Filipits[2], Giannantonio Cibin[3], Bernhard K. Keppler[4] & Annette Rompel[1]*

[1] Universität Wien, Fakultät für Chemie, Institut für Biophysikalische Chemie, Althanstraße 14, 1090 Wien, Austria; http://www.bpc.univie.ac.at

*Correspondence to annette.rompel@univie.ac.at

[2] Medizinische Universität Wien, Institut für Krebsforschung, “Comprehensive Cancer Center” und Forschungsplattform “Translational Cancer Therapy Research”, Borschkegasse 8a, 1090 Wien, Austria

[3] Diamond Light Source, Didcot, OX11 0DE, United Kingdom

[4] Universität Wien, Fakultät für Chemie, Institut für Anorganische Chemie und Forschungsplattform “Translational Cancer Therapy Research”, Währinger Straße 42, 1090 Wien, Austria

Table

Supplementary Table 1. Cl K-edge pre-edge fits of Ru–Cl compounds; FWHM: full width at half maximum.

Compound	Ru first shell	Ru-Cl bond length [Å]	Ref.	energy (eV)[a]	amplitude	FWHM	area	total area
**3**	$Ru^{III}O_2Cl_3N$	2.372,	[1]	2821.3	0.120±0.027	0.485±0.038	0.082±0.048	2.012±0.038
		2.359,		2821.8	1.092±0.018	1.228±0.034	1.930±0.011	
		2.349						
**4**	$Ru^{III}OCl_3N_2$	2.338,	[2]	2821.3	0.116±0.009	0.519±0.024	0.073±0.042	2.005±0.034
		2.338,		2822.0	0.788±0.012	1.560±0.019	1.932±0.020	
		2.318						
**5**	$Ru^{III}Cl_2N_4$	2.334,	[3]	2820.6	0.140±0.024	0.834±0.007	0.154±0.011	1.868±0.019
		2.328		2822.4	0.452±0.032	1.196±0.054	1.714±0.026	
**6**	$Ru^{III}Cl_3N_3$	2.343,	[4]	2820.8	0.337±0.024	0.851±0.017	0.378±0.004	1.965±0.014
		2.342,		2822.4	0.984±0.018	1.533±0.024	1.587±0.064	
		2.340						
**7**	$Ru^{III}Cl_4N_2$	2.359,	[5]	2821.1	0.104±0.004	0.880±0.011	0.134±0.014	2.176±0.009
		2.632,		2822.3	0.674±0.034	1.174±0.038	2.042±0.004	
		2.363,						
		2.372						
**8**	$Ru^{III}SCl_3N_2$	2.344,	[6]	2820.5	0.146±0.037	0.680±0.045	0.116±0.009	1.817±0.010
		2.347,		2822.1	0.801±0.031	1.309±0.009	1.701±0.012	
		2.360						
**11**	$Ru^{III}S_3Cl_3$	2.368,	[7]	2820.7	0.309±0.023	1.019±0.024	0.399±0.029	1.903±0.037
		2.369,		2822.1	0.789±0.009	1.315±0.039	1.404±0.044	
		2.372						
**12**	$Ru^{II}N_4Cl_2$	2.424,	[3]	2823.5	0.654±0.028	1.087±0.029	1.045±0.057	1.045±0.057
		2.412						
**13**	$Ru^{II}S_2Cl_2N_2$	2.430,	[6]	2823.4	0.664±0.024	0.840±0.032	0.989±0.012	0.989±0.012
		2.430						

[a] pre-edge energy at maximum

Figures

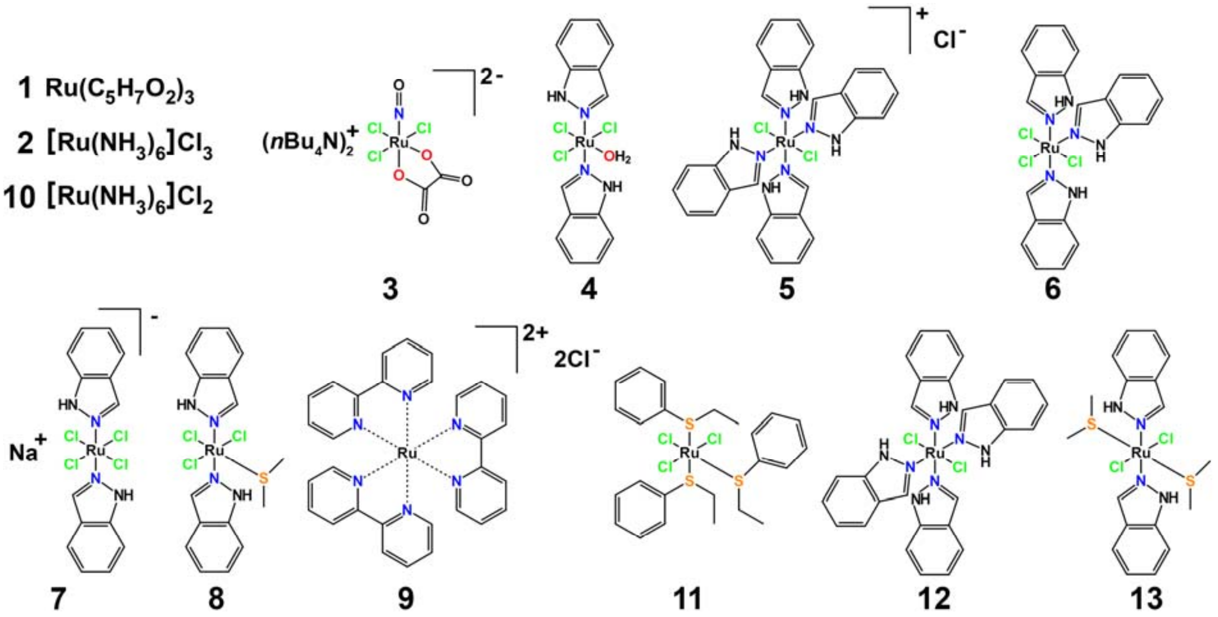

Supplementary Figure 1. Structures (**1**-**13**) of the Ru model compounds with first coordination shell atoms in color. They are presented in order of the edge energy (high to low) based on the first maximum in the first derivative at the Ru L_2-edge. Compounds **1**-**9** contain Ru^{III} and compounds **10**-**13** contain Ru^{II}. Compounds **1**-**13**: Ruthenium(III) acetylacetonate (**1**, with first coordination shell $Ru^{III}O_6$, Sigma Aldrich, CAS 14284-93-6, 97%)[8], hexammineruthenium(III) trichloride (**2**, $[Ru(NH_3)_6]Cl_3$, $Ru^{III}N_6$, Sigma Aldrich, CAS 14282-91-8, 99%)[9], $(n\text{Bu}_4N)_2[RuCl_3(ox)(NO)]$ (**3**, GABU527, $Ru^{III}Cl_3NO_2$)[1], *mer*,*trans*-aquatrichloridobis(indazole)ruthenium(III) (**4**, KASC003, $Ru^{III}Cl_3N_2O$)[2], *trans*,*trans*-dichloridotetrakis(indazole)ruthenium(III) chloride (**5**, GABU129, $Ru^{III}Cl_2N_4$)[3], *mer*-trichloridotris(indazole)ruthenium(III) (**6**, GUPL328, $Ru^{III}Cl_3N_3$)[4], sodium *trans*-[tetrachloridobis(1*H*-indazole)ruthenate(III)] (**7**, NKP-1339, $Ru^{III}Cl_4N_2$)[5], *mer*,*trans*-trichlorido(dimethylsulfide)bis(indazole)ruthenium(III) (**8**, FLAN005, $Ru^{III}Cl_3N_2S$)[6], tris(bipyridine)ruthenium(II) chloride (**9**, $Ru^{II}N_6$, Sigma Aldrich, CAS 50525-27-4, 99.95%)[10], hexammineruthenium(II) dichloride (**10**, $[Ru(NH_3)_6]Cl_2$, $Ru^{II}N_6$, Sigma Aldrich, CAS 15305-72-3, 99.9%)[9], *mer-mer*-trichloridotris(ethylphenylsulfide)ruthenium(III) (**11**, FLAN006, $Ru^{III}Cl_3S_3$)[7], *trans*,*trans*-dichloridotetrakis(indazole)ruthenium(II) (**12**, GABU128, $Ru^{II}Cl_2N_4$)[3] and *trans*,*trans*,*trans*-dichloridobis(dimethylsulfide)bis(indazole)ruthenium(II) (**13**, FLAN004, $Ru^{II}Cl_2N_2S_2$)[6].

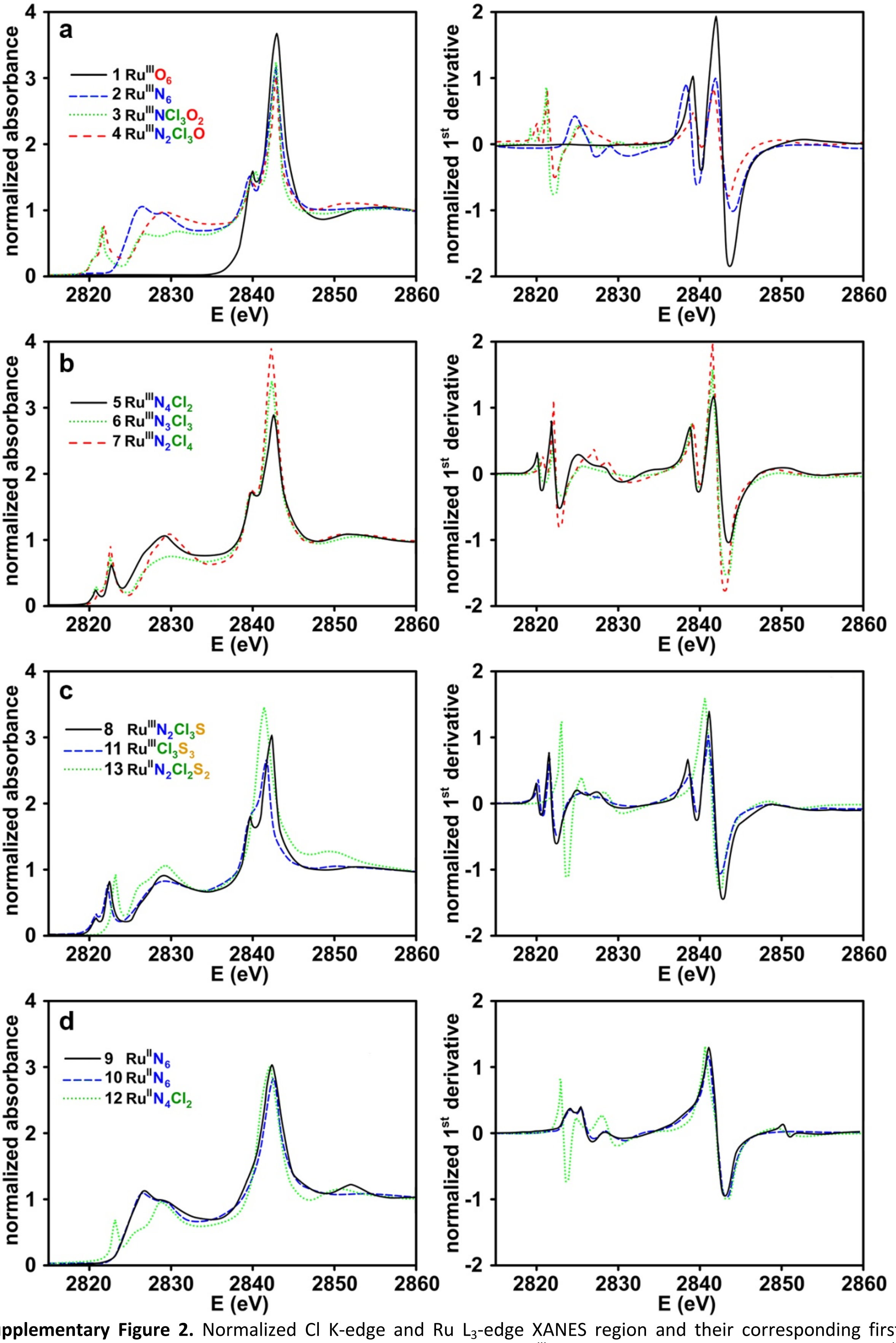

Supplementary Figure 2. Normalized Cl K-edge and Ru L_3-edge XANES region and their corresponding first derivatives of model compounds **1**-**13**. Model compounds with Ru^{III} containing O and N are shown in (a), Ru^{III} compounds containing N and Cl in (b), Ru compounds containing S in (c) and Ru^{II} compounds in (d).

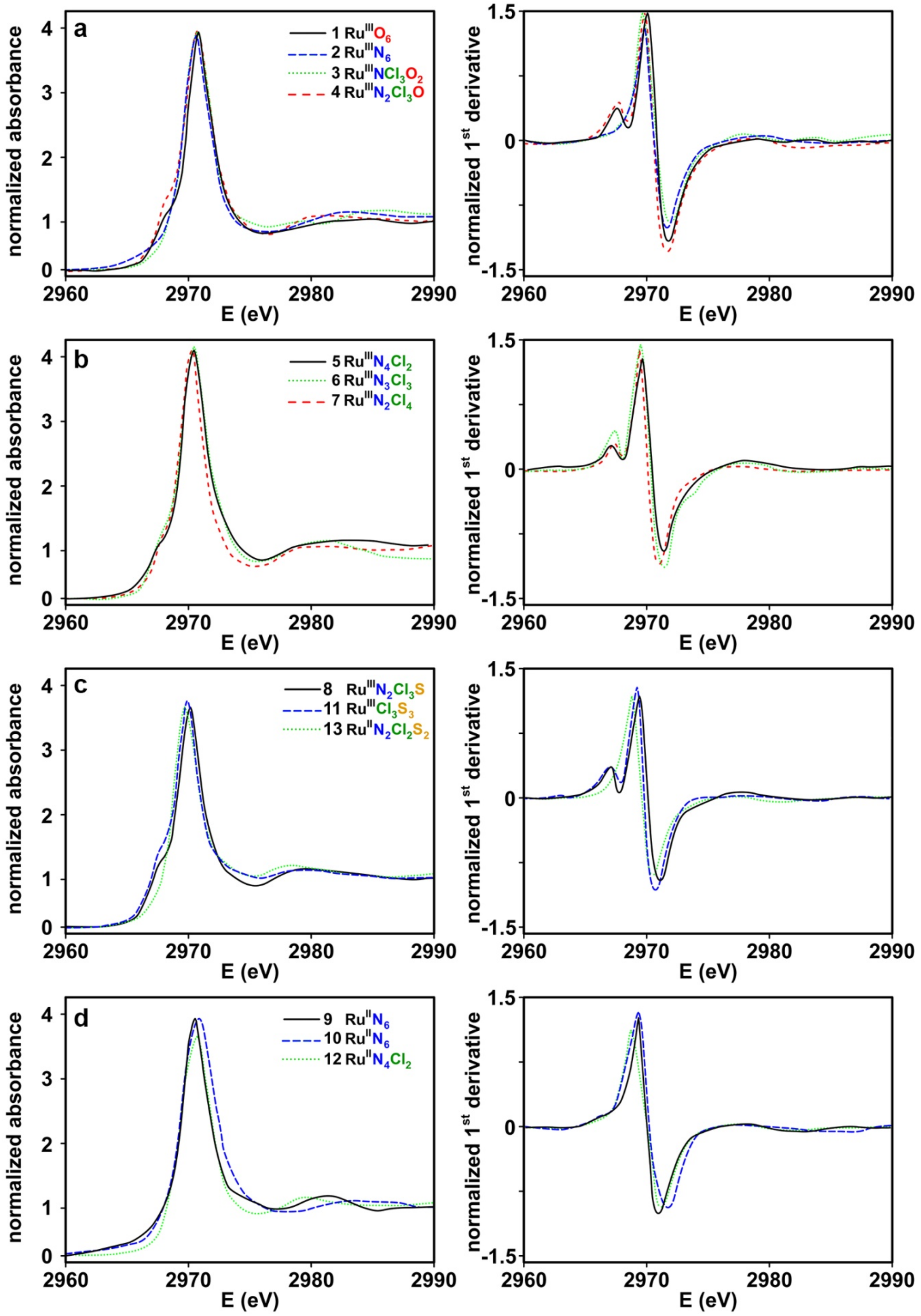

Supplementary Figure 3. Normalized Ru L_2-edge XANES region and their corresponding first derivatives of model compounds **1**-**13**. Model compounds with Ru^{III} containing O and N are shown in (a), Ru^{III} compounds containing N and Cl in (b), Ru compounds containing S in (c) and Ru^{II} compounds in (d).

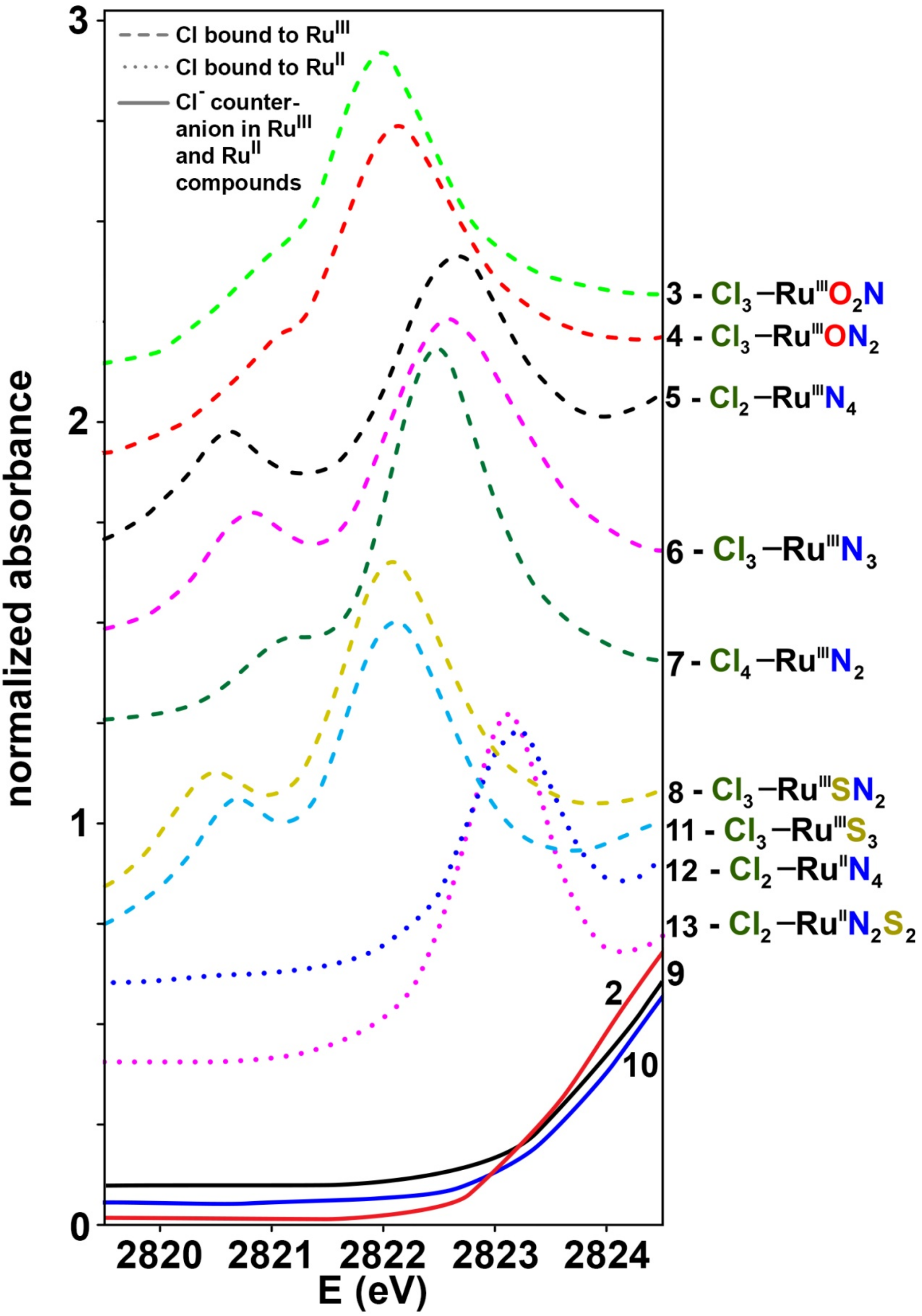

Supplementary Figure 4. Pre-edge peak region of the normalized Cl K-edge spectra for Cl containing model compounds. The model compounds are shown in three different groups; Cl covalently bound to Ru^{III} (dashed lines), Cl covalently bound to Ru^{II} (dotted lines) and Cl$^-$ counteranion present in Ru^{II} and Ru^{III} model compounds (solid lines). Spectra are plotted with an arbitrary vertical shift.

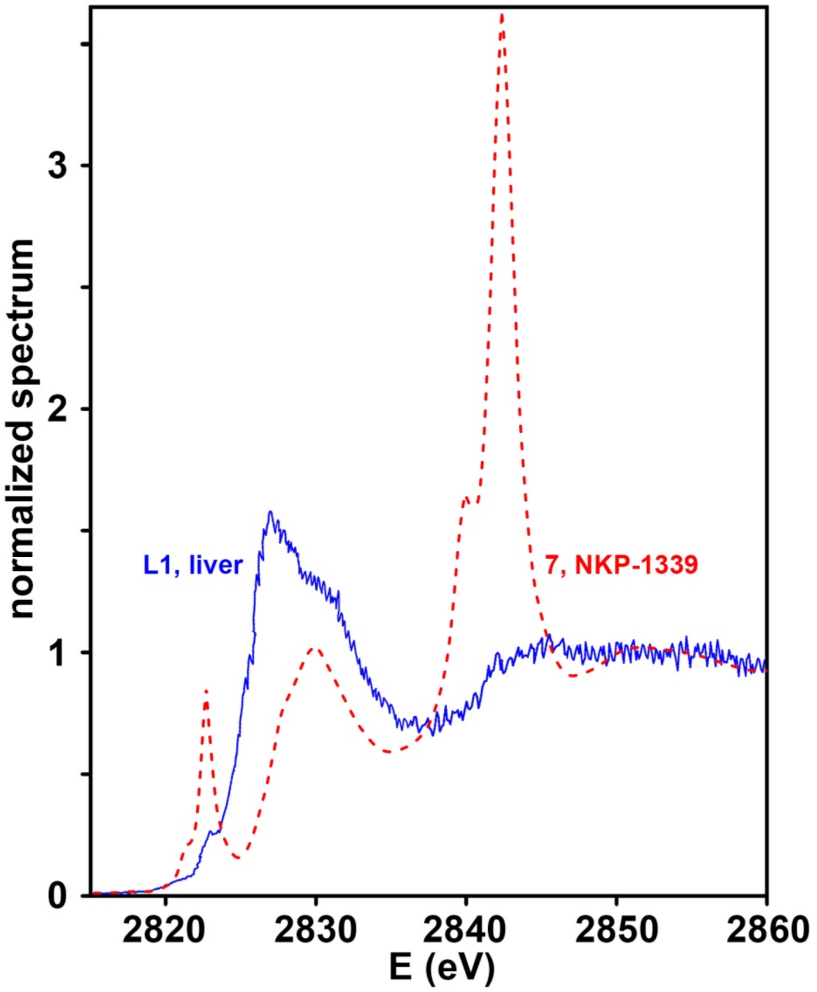

Supplementary Figure 5. Normalized Cl K-edge and Ru L_3-edge of samples L1 and NKP-1339 (measured in boron nitride).

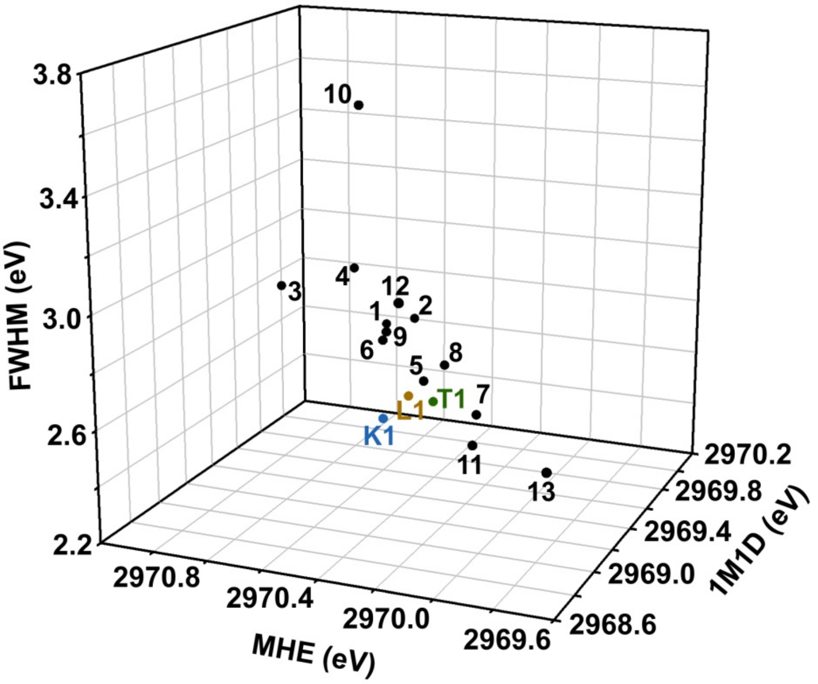

Supplementary Figure 6. 3D XANES plot based on the edge energies of the 1st maximum in the first derivative (1M1D), maximum height (MHE) and the full width at half maximum (FWHM).
